# Melanin-Like Pigment Synthesis by Soil *Bacillus weihenstephanensis* Isolates from Northeastern Poland

**DOI:** 10.1371/journal.pone.0125428

**Published:** 2015-04-24

**Authors:** Justyna M. Drewnowska, Monika Zambrzycka, Beata Kalska-Szostko, Krzysztof Fiedoruk, Izabela Swiecicka

**Affiliations:** 1 Department of Microbiology, Institute of Biology, University of Bialystok, Bialystok, Poland; 2 Department of Physicochemical Analysis, Institute of Chemistry, University of Bialystok, Bialystok, Poland; 3 Department of Microbiology, Medical University of Bialystok, Bialystok, Poland; 4 Laboratory of Applied Microbiology, University of Bialystok, Bialystok, Poland; Belgian Nuclear Research Centre SCK•CEN, BELGIUM

## Abstract

Although melanin is known for protecting living organisms from harmful physical and chemical factors, its synthesis is rarely observed among endospore-forming *Bacillus cereus sensu lato*. Here, for the first time, we reported that psychrotolerant *Bacillus weihenstephanensis* from Northeastern Poland can produce melanin-like pigment. We assessed physicochemical properties of the pigment and the mechanism of its synthesis in relation to *B*. *weihenstephanensis* genotypic and phenotypic characteristics. Electron paramagnetic resonance (EPR) spectroscopy displayed a stable free radical signal of the pigment from environmental isolates which are consistent with the commercial melanin. Fourier transform infrared spectroscopy (FT-IR) and physicochemical tests indicated the phenolic character of the pigment. Several biochemical tests showed that melanin-like pigment synthesis by *B*. *weihenstephanensis* was associated with laccase activity. The presence of the gene encoding laccase was confirmed by the next generation whole genome sequencing of one *B*. *weihenstephanensis* strain. Biochemical (API 20E and 50CHB tests) and genetic (Multi-locus Sequence Typing, 16S rRNA sequencing, and Pulsed-Field Gel Electrophoresis) characterization of the isolates revealed their close relation to the psychrotrophic *B*. *weihenstephanensis* DSMZ 11821 reference strain. The ability to synthesize melanin-like pigment by soil *B*. *weihenstephanensis* isolates and their psychrotrophic character seemed to be a local adaptation to a specific niche. Detailed genetic and biochemical analyses of melanin-positive environmental *B*. *weihenstephanensis* strains shed some light on the evolution and ecological adaptation of these bacteria. Moreover, our study raised new biotechnological possibilities for the use of water-soluble melanin-like pigment naturally produced by *B*. *weihenstephanensis* as an alternative to commercial non-soluble pigment.

## Introduction

Melanin is a heterogenous and polymeric pigment found in many Prokaryote and Eukaryote organisms. Melanin production has been considered to be of a great significance, especially with regard to microorganisms in which it has been often associated with virulence in the host and survival advantage in the environment [1]. For instance, melanin synthesized by free-living microorganisms absorbs a broad spectrum of electromagnetic radiation, from visible light to ionizing radiation [2] protects their producers from reactive oxygen forms [3], heavy metals toxicity [4], and extreme temperatures [5]. These properties of melanin make it an attractive biomaterial used as an ingredient in sunscreens [6], wool fabric dyes [7] and *Bacillus thuringiensis*-based biopesticides for UV protection [8]. Moreover, melanin protects pathogens against immune responses of a microbe's host [9], and appears to play an important role in the development of symbiosis between soil bacteria and plants [10]. In most organisms, melanin production starts with an enzymatic reaction of L-tyrosine via L-DOPA (L-3,4-dihydroxyphenylalanine) to DOPA-quinone, which involves enzymes such as tyrosinase or tyrosine hydroxylase [11, 12]. In some fungi, such as *Cryptococcus neoformans*, the conversion of L-DOPA to DOPA-quinone occurs with the participation of laccase [13]. Subsequently, a series of non-enzymatic reactions leads to the formation of black or brown eumelanin or orange-yellow pheomelanin [11–13]. The alternative pathway, involving degradation of L-tyrosine to homogentisic acid (HGA) which can polymerize to brown pyomelanin, also has been observed in some fungi and bacteria, such as *Aspergillus fumigatus*, *Legionella pneumophila*, *Pseudomonas aeruginosa* or *Vibrio cholerae* [14–16]. Moreover, dark green pigment synthetized by DHN- or HPQ-melanin pathway was characterized in fungi [17] and bacteria [18], respectively.

*Bacillus weihenstephanensis* is a psychrotrophic Gram-positive aerobic or facultative anaerobic bacterium [19], commonly present in food matrices [20] and soil [21]. This bacterium belongs to the *Bacillus cereus* group (*B*. *cereus sensu lato*) which includes seven other species commonly occurring in different environments and being extremely important for medical and economic reasons [21, 22]. The most extensively studied are (i) *Bacillus anthracis*, etiologic agent of anthrax [23], (ii) *Bacillus cereus*, an opportunistic pathogen occasionally involved in foodborne illnesses [24], and (iii) *B*. *thuringiensis*, entomopathogen universally used as a biopesticide [25]. Much less is known about other members of the group, *Bacillus mycoides* and *Bacillus pseudomycoides*, characterized by rhizoidal growth on solid media, antifungal activity and stimulation of plants’ growth [26]. *B*. *cereus s*.*l*. also contains recently described thermotolerant *Bacillus cytotoxicus* [27] and *Bacillus toyonensis*, a probiotic organisms used in animal feed [28]. Although it has been established that the *B*. *cereus* group members are versatile producers of secondary metabolites, such as antimicrobial substances [29], extracellular enzymes [30] or fluorescent pigments [31], little is known about strains synthesizing melanin. Only two melanin-positive wild strains, *B*. *thuringiensis* subsp. *dendrolimus* L-7601 [32] and *B*. *thuringiensis* subsp. *kurstaki* CCTCC AB90010 [33], have been described. However, melanin producers among *B*. *weihenstephanensis* have not been reported so far. In several studies blackish-brown pigment production in *B*. *cereus s*.*l*. has been obtained through chemical mutagenesis [34] or genetic modification [35]. Nevertheless, the mechanism of melanin production among these species is poorly understood.

In this work, for the first time, we report the natural production of water-soluble melanin-like pigment by soil *B*. *weihenstephanensis* isolates from Northeastern Poland. We also assessed physicochemical properties of melanin-like pigment synthesized by these bacilli and proposed the mechanism of its synthesis. The unique properties of environmental strains prompted us to conduct phenotypic, genotypic and phylogenetic analysis, which gave insight into their evolution.

## Materials and Methods

### Bacterial strains

A collection of about 950 *B*. *cereus s*.*l*. isolates from soil, arthropods, and foodstuffs. Soil samples were obtained from Bialowieza National Park (N 52° 72’, E 23° 84’), Biebrza National Park (N 53° 36’, E 22° 56^’^), and agricultural land in Jasienowka (N 52° 30’, E 22° 58), Northeastern Poland. All samples from the parks were collected with consent according to Nature Conservation Act adopted on 16 April, 2004 by Polish Parliament (Parliament Diary 2004, No. 92: 880). The owner of the farm also permitted collection of soil samples for our study. The strains were isolated during previous studies [20, 36, 37]. The field studies did not involve endangered or protected species.

The isolates were screened by culturing on different media as Luria-Bertani (LB) agar, LB broth, nutrient agar, nutrient broth, and sporulation medium T3 (0.3% tryptone, 0.2% tryptose, 0.15% yeast extract, 0.05 M sodium phosphate pH 6.8, 0.0005% MgCl_2_) in order to select producers of a blackish-brown pigment diffusing into the medium.The collection included bacilli identified in previous studies as *B*. *cereus*, *B*. *thuringiensis*, *B*. *mycoides/B*. *pseudomycoides*, and *B*. *weihenstephanensis*. *B*. *cereus* ATCC 10987 and ATCC 14579 (American Type Culture Collection), *B*. *thuringiensis* HD1, HD73, HD567, and HD867 (*Bacillus* Genetic Stock Center, Ohio State University), and *B*. *weihenstephanensis* DSMZ 11821 (German Collection of Microorganisms and Cell Cultures), were used as references during phenotypic and genotypic tests of the melanin-positive isolates tested in this study.

### Psychrotolerance of pigment producers

The blackish-brown pigment-positive isolates were tested for the ability to grow at 7°C in LB broth and on nutrient agar plates. Then their psychrotrophic potential was confirmed in PCR by investigating the presence of specific signatures of *cspA* and 16S rDNA genes [19] tested with primers designed by Bartoszewicz et al. [20] and Lechner et al. [19], respectively. PCRs were carried out in the Veriti 96-Well thermal cycler (Applied Biosystems, Foster City, USA), followed by the analysis of amplicons in the QIAxcel capillary electrophoresis system (Qiagen GmbH, Hilden, Germany). Genes were sequenced using Big Dye Terminator Cycle Sequencing Kit (Applied Biosystems) and automated sequencer ABI3500 (Applied Biosystems). A phylogenetic tree of 16S rDNA partial homologs (1463 bp) was constructed with MEGA6 program using the Neighbor-Joining (NJ) method with the branch quality evaluated including 1,000 replicates bootstrap test [38].

### Biochemical characterization

Biochemical properties of isolates and references were verified with API 50CHB and API 20E system (bio-Mérieux S.A., Mercy l'Etoile, France), according to the manufacturer’s procedure and as recommended by Swiecicka and de Vos [39]. Biochemical similarity was calculated using the simple matching coefficient (SMC) and clustered with unweighted pair-group average linkage algorithm (UPGMA) performed with the NTSys ver. 2.02 g program (Exeter Software, E. Setauket, NY, USA) as described previously [39].

### Phylogeny based on multi-locus sequence typing (MLST)

Nucleotide sequences of seven housekeeping genes (*glpF*, *gmk*, *ilvD*, *pta*, *pur*, *pycA*, *tpi*) deposited in the MLST database (http://pubmlst.org/bcereus/) during previous studies [37] and corresponding sequences of reference strains also available in the database, were used to construct a phylogenetic tree with MEGA6 program using the Neighbor-Joining (NJ) method. The branch quality was evaluated using 1,000 replicates bootstrap test [38].

### Pulsed-field gel electrophoresis of genomic DNA

Overnight cultures of strains were grown in LB and centrifuged at 4°C and 2,500 x g for 15 min and resuspended in SE buffer (10 mM NaCl, 30 mM EDTA, pH 7.5) to an OD_590_ of approximately 2.0. Genomic DNA plugs were prepared by mixing bacterial suspension with 2% LMP agarose (Sigma-Aldrich, Poznan, Poland) at 1:1 ratio and placed into slots of a plug mold (Bio-Rad, Hercules, CA, USA). After solidification, plugs were treated according to Gaviria Rivera and Priest [40], DNA was digested with 30 U of NotI restrictase (MBI Fermentas, Vilnius, Lithuania) and electrophoresed in the CHEF-MAPPER System (Bio-Rad) following the protocol of Swiecicka et al. [41]. PFG Lambda Ladders and PFG Yeast chromosomes from New England BioLabs were used as markers. Gels were stained with ethidium bromide solution (1 μg ml^-1^) and visualized in ChemiDOC XRS System (Bio-Rad).

### Extraction, purification, and chemical characteristics of melanin-like pigment

The pigment was isolated from the four most productive strains: JAS 39/1, JAS 81/4, JAS 83/3, and JAS 86/1. Isolates were inoculated into 200 ml of nutrient broth and incubated at 30°C on rotary shaker at 180 rpm for 96 h until the liquid medium became dark-brown. When the cell mass was removed, the supernatant was acidified by lowering a pH to 2.0 using 1N HCl and incubated at room temperature for one week, followed by boiling of the suspension for 1 h and centrifuging at 14,000 x g for 15 min. The pigment pellet was washed with ethanol as described by Sajjan et al. [42]. The modified method of Fava et al. [43] was used to perform chemical analyses of the extracted melanin-like pigment. The pigment solubility was checked in deionized water, 1 N NaOH, 1 N HCl, acetone, benzene, chloroform, ethanol, and phenol. In addition, the reactions of melanin with 30% hydrogen peroxide (H_2_O_2_), 1% iron (III) chloride (FeCl_3)_, and 5% sodium hydrosulfite (Na_2_S_2_O_4_), were tested. Synthetic melanin (Sigma-Aldrich, Cat. No M8631) was used as a reference.

### Electron paramagnetic resonance (EPR) measurement

The electron paramagnetic resonance measurement for the tested melanin-like pigments and synthetic melanin were performed with the X-band (9,3 GHz) EPR spectrometer (Radiopan, Poznan, Poland) and the Rapid Scan Unit (Jagmar, Krakow, Poland). Each sample was placed in a thin-walled glass tube free of paramagnetic impurities. Microwave frequency was obtained by the MCM101 Recorder (Eprad, Poznan, Poland) at magnetic modulation of 100 kHz. The total microwave power of the klystron was 70 mW. The numerical acquisitions of the first-derivative EPR spectra were done at low microwave power of 11 mW. The spectroscopic programs SWAMP (Jagmar) and LabVIEW 8.5 (National Instruments) were used.

### Fourier transform infrared (FT-IR) spectroscopy

To quantify the extracted melanin-like pigment, IR spectra were recorded by Nicolet 6700 infrared spectrometer (Thermo Fisher Scientific, Warsaw, Poland). A small amount of pigment was placed on a diamond window of the spectrometer, and a measurement was done in reflection mode, at a room temperature, by summary of 32 scans with a resolution of 4 cm^-1^. The available spectra range was 400–4000 cm^-1^. Synthetic melanin (Sigma-Aldrich, Cat. No M8631) was used as a standard.

### Mechanisms of pigment production

LB was inoculated with melanin-positive *B*. *cereus s*.*l*. isolates and incubated at 30°C on a rotary shaker at 180 rpm for 24 h. After the preincubation, arginine [44] or kojic acid [45] as tyrosinase inhibitors were added to a final concentration of 0.01–0.5 mM and 10–100μg/ml, respectively. As a laccase inhibitor, sodium azide was added to a final concentration of 0.01–0.2 mM [46]. In addition, the sulcotrione [16] and tricyclazole [45] were used for 4-hydroxyphenylpyruvate dioxygenase (4-HPPD) and hydroxynaphthalene reductase inhibition, respectively, to confirm/exclude the HGA or DHN pathways of melanin synthesis. Then, cultures were incubated at 30°C on the rotary shaker at 180 rpm for 96 h till the dark color occurred in the control culture.

### Next generation whole genome sequencing

Genomic DNA of *B*. *cereus* strain JAS 83/3, one of the most melanin-productive isolate, was extracted in the QIAcube automat (Qiagen) using the DNeasy Blood and Tissue Kit (Qiagen) with a protocol for Gram-positive bacteria. The draft genome sequence of the strain was determined using an Ion Torrent PGM sequencer (Applied Biosystems) using the Ion 316 chip with 200-bp shotgun sequencing, according to the manufacturer's instructions. The annotation was performed using best-placed reference protein set GeneMarkS+ provided by the National Center for Biotechnology Information (NCBI).

### GenBank accession numbers

The whole sequence of *B*. *weihenstephanensis* JAS 83/3 obtained by shotgun sequencing is under Accession Number: JNLY00000000 (BioProject: *Genome sequencing of melanin-positive Bacillus weihenstephanensis strain* JAS 83/3; NCBI; PRJNA246580). We also deposited three 16S rRNA homologs of *B*. *weihenstephanensis* JAS 83/3 (KP006648), *B*. *thuringiensis* HD567 (KP006649), and *B*. *thuringiensis* HD867 (KP006650). Remaining sequences of 16S rRNA were available under accession number: NR_024697 (*B*. *weihenstephanensis* DSMZ 11821), BTK_r29390 (*B*. *thuringiensis* HD1), HD73_r36 (*B*. *thuringiensis* HD73), BC0007 (*B*. *cereus* ATCC 14579), and BCE5759 (*B*. *cereus* ATCC 10987).

## Results

### Melanin-like pigment synthesis among *B*. *cereus s*.*l*. from Northeastern Poland is very rare and is restricted to *B*. *weihenstephanensis*

**Fig 1:**
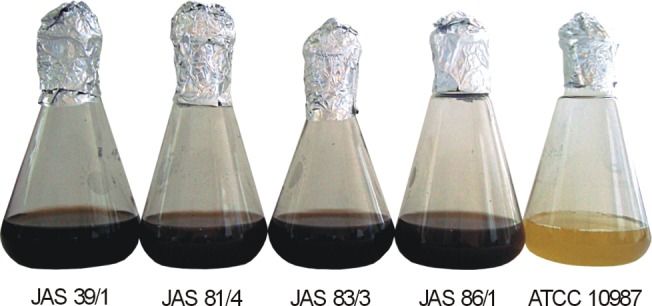
Melanin-like pigment production in Luria-Bertoni broth by *B*. *weihenstephanensis*. *B*. *cereus* ATCC 10987 was used as the negative control.

In order to study the production of melanin-like pigment by *B*. *cereus s*.*l*., a large collection of about 950 strains identified as *B*. *cereus*, *B*. *thuringiensis*, *B*. *mycoides/B*. *pseudomycoides*, and *B*. *weihenstephanensis*, was screened by culturing on different growth media (LB, nutrient agar, T3 agar, LB and nutrient broths) and observed for the blackish-brown pigment diffused in the medium. Although the origins of the strains were diverse (soil, arthropods, mammals, and foodstuffs), the pigment-positive bacilli were found only among six *B*. *weihenstephanensis* isolates from soil samples obtained from Jasienowka farm (JAS 39/1, JAS 81/4, JAS 83/3, JAS 86/1) and Bialowieza National Park (BPN 08/1, BPN 08/4) in Northeastern Poland. However, the production of pigment by isolates from the park was low and observed only on NA plates. In contrast, the farm isolates synthesized the pigment at high quantity both on NA plates and in LB and nutrient broths (Fig 1). These bacilli grew well at 7°C. Their psychrotrophic adaptation was confirmed by the presence of unique nucleotide motifs: (i) ^4^ACAGTT^9^ in the *cspA* gene encoding the major cold shock protein, and (ii) ^1002^TCTAGAGATAGA^1013^ in the 16S rDNA (S1 Table).

**Fig 2:**
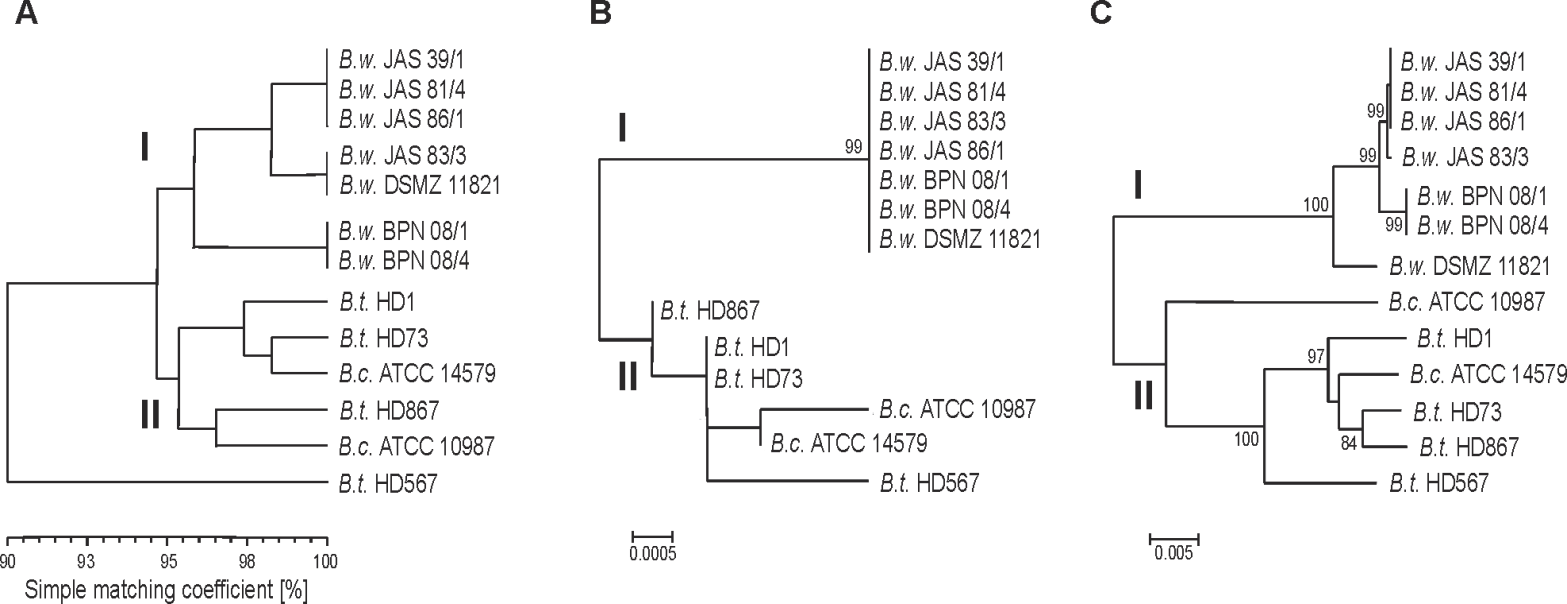
The phenotypic similarity and phylogeny among *B*. *weihenstephanensis* producing melanin-like pigment and reference strains. Comparisons between strains based on biochemical API 50CH and API 20E tests were made using simple matching coefficient and clustered with the UPGMA algorithm (A). Phylogenetic trees were constructed based on 16S rDNA gene (B) and seven concatenated housekeeping loci (MLST) (C) using the Neighbor-Joining (NJ) method implemented in MEGA6 software, where branch quality was evaluated using 1,000 replicates bootstraps.

Biochemical properties of pigment-positive and reference strains were tested using 60 different tests available with the API system (see summary in S1 Table). In short, all environmental isolates produced acetoin, liquefied gelatin and fermented 11 of 49 carbohydrates. Variable results were received for acid production from cellobiose and sucrose as well as for arginine dihydrolysis. Biochemical profiles of reference strains used in the study were similar to these found for the isolates (S1 Table). A dendrogram based on biochemical properties and calculated using the simple matching coefficient and UPGMA algorithm (Fig 2A), showed high similarity between soil isolates and reference *B*. *weihenstephanensis* DSMZ 11821. *B*. *cereus* ATCC 10987, *B*. *cereus* ATCC 14579, *B*. *thuringiensis* HD1, *B*. *thuringiensis* HD73, and *B*. *thuringiensis* HD867 reference strains grouped in a second cluster. *B*. *thuringiensis* HD567, known for anti-mosquito properties [47], differed biochemically from other strains and made cluster III.

**Fig 3:**
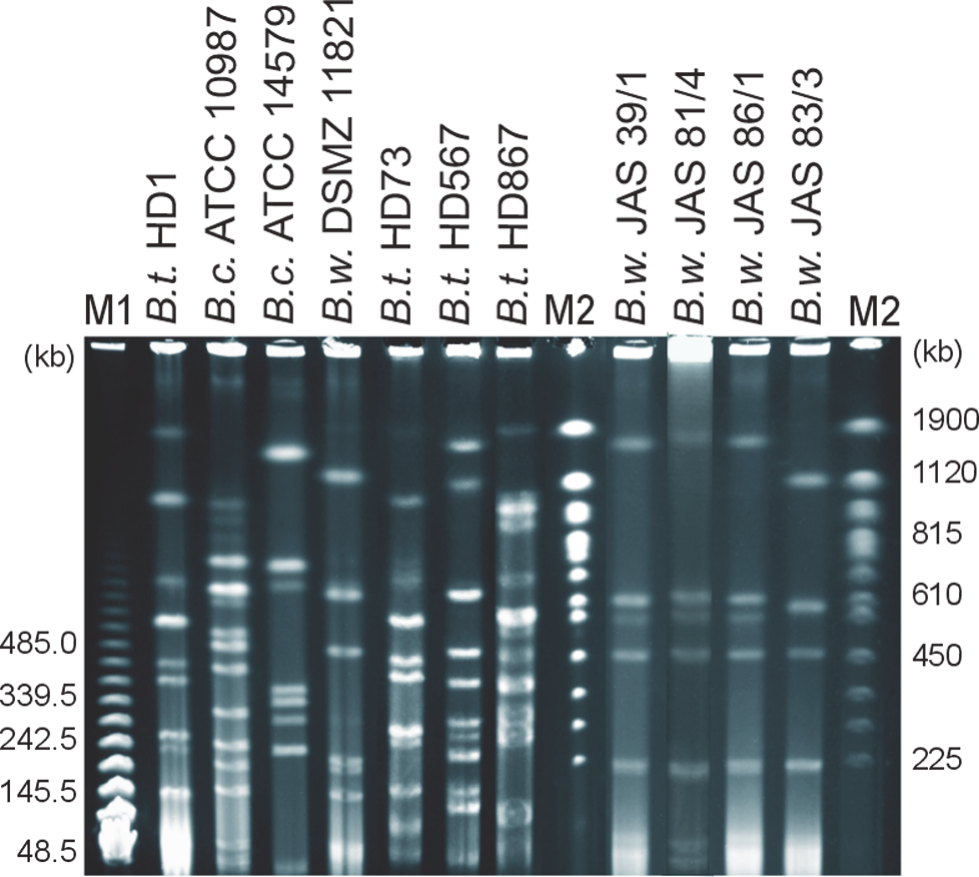
PFGE fingerprints of melanin-positive *B*. *weihenstephanensis* isolates and reference strains. Genomic DNA was digested using NotI. M1, PFG Lambda Ladders; M2, PFG Yeast chromosomes. The values on the left and right are molecular weight marker in kb.

Comparative analysis of 16S rRNA confirmed close relation between melanin-positive isolates and *B*. *weihenstephanensis* DSMZ 11821 (Fig 2B). On the phylogenetic tree, isolates grouped together with DSMZ 11821, while other references gathered in the second cluster. Phylogeny based on multi-locus sequence typing also revealed two genetic clusters in the Neighbour-Joining dendrogram (Fig 2C). Soil isolates grouped in cluster I with DSMZ 11821, whereas other references were classified into cluster II. Pulsed-field gel electrophoresis (PFGE) confirmed the clonality of *B*. *weihenstephanensis* JAS 39/1, JAS 81/4 and JAS 86/1 (Fig 3), while *B*. *weihenstephanensis* BPN 08/1 and BPN 08/4 were not typeable by PFGE (profiles has not been achieved). Pulsed-field fingerprints of isolates do not overlap with PFGE profiles of reference strains.

### Melanin-like pigment synthesized by soil *B*. *weihenstephanensis* isolates had phenolic character

**Fig 4:**
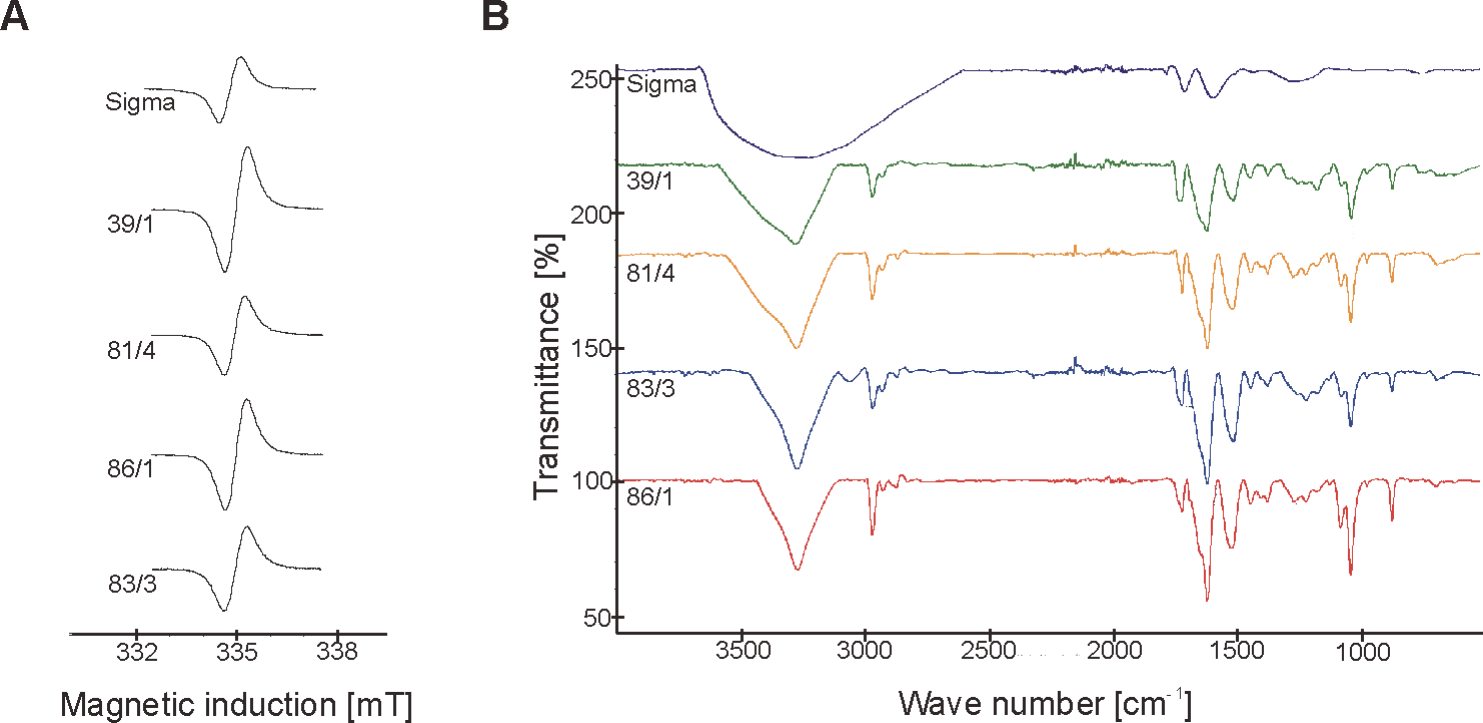
EPR (A) and FT-IR (B) spectra of commercial melanin and pigment obtained from *B*. *weihenstephanensis* isolates.

EPR spectra of natural pigment produced by environmental strains of *B*. *weihenstephanensis* from the farm (isolates from Bialowieza National Park did not produce enough pigment to analyse) were similar to the EPR signal of the synthetic melanin and all revealed broad curves around 335 mT (Fig 4A). In addition, FT-IR spectroscopy of dark particles demonstrated similar spectra to synthetic one (Fig 4B). A broad band centered around 3268–3278 cm^-1^ for each studied pigment was observed, which is associated with-OH stretching. Furthermore, all samples had absorbance peaks in the 1511–1729 cm^-1^ area, due to the bonding vibration of C = C and C = O aromatic ring stretching, and double bonds in COOH. In contrast to standard melanin, *in vitro* synthesized melanin had signals in 2926–2970 cm^-1^ area indicating the presence of saturated carbon, as well as around 1045 cm^-1^ and 1220 cm^-1^, what corresponds to carbonyl, alcoholic or phenolic groups, respectively. Detailed information on functional groups found in melanin-like pigment synthesized by environmental isolates and synthetic melanin is given in Table 1.

**Table 1:** Functional groups of melanin-like pigment produced by soil *B*. *weihenstephanensis* isolates and commercial melanin obtained from Fourier Transform Infrared spectroscopy.

Assignment	Wave number [cm^-1^]					Reference
	*Bw* JAS 39/1	*Bw* JAS 81/4	*Bw* JAS 83/3	*Bw* JAS 86/1	Commercial melanin	
-OH, stretching	3274	3278	3268	3271		[48]
Aliphatic-CH, stretching	2967	2970	3069	2964		[49]
			2964	2929		
			2926			
-COOH	1729	1723	1726	1723	1707	[49]
Aromatic ring C = C and C = O, stretching	1622	1615	1622	1619	1603	[49]
-COOH	1511	1517	1511	1524		[49]
C-H, bonding	1448	1451	1448	1448	1438	[48]
-COO, symmetric stretching	1375	1378	1375	1375		[50]
C-H, deformation	1264	1277		1277	1283	[51]
C = O	1178	1220	1220			[49]
C-O, close to aromatic ring	1042	1045	1045	1045		[51]
N-H, O-H, bending	874	874	874	871	757	[48]

Chemical properties of melanin-like pigment produced by soil *B*. *weihenstephanensis* isolates and of synthetic melanin were also similar (S2 Table). Pigments were soluble in alkaline solution (1N NaOH) and phenol, but were insoluble in ethanol, acetone, chloroform, and benzene. Moreover, dissolved pigments and synthetic melanin precipitated in hydrochloric acid (1N HCl) and ferric chloride (1% FeCl_3_), and were decolorized by hydrogen peroxide (30% H_2_O_2_), as well as by sodium hydrosulfite (5% Na_2_S_2_O_4_). The only feature which differed natural pigment and synthetic melanin was the solubility in water, observed only for pigments from *B*. *weihenstephanensis*.

### Next generation whole genome sequencing and a set of biochemical tests indicate that melanin-like pigment synthesis by *B*. *weihenstephanensis* could be associated with the laccase activity

Shotgun sequencing of *B*. *weihenstephanensis* strain JAS 83/3 was performed to identify genes associated with melanin-like pigment synthesis. We found genes encoding enzymes potentially involved in the synthesis of melanin such as: laccase, phenylalanine 4-monooxygenase, pterin-4-alpha-carbinolamine dehydratase, aromatic amino acid aminotransferase, and 4-hydroxyphenylpyruvate dioxygenase. It is worth adding that JAS 83/3 genome consisted of 5,902,407 bp and contained 5,362 coding sequences (CDSs), 347 pseudogenes, and 99 RNA operons (seven rRNA, 91 tRNA and one ncRNA).

**Fig 5:**
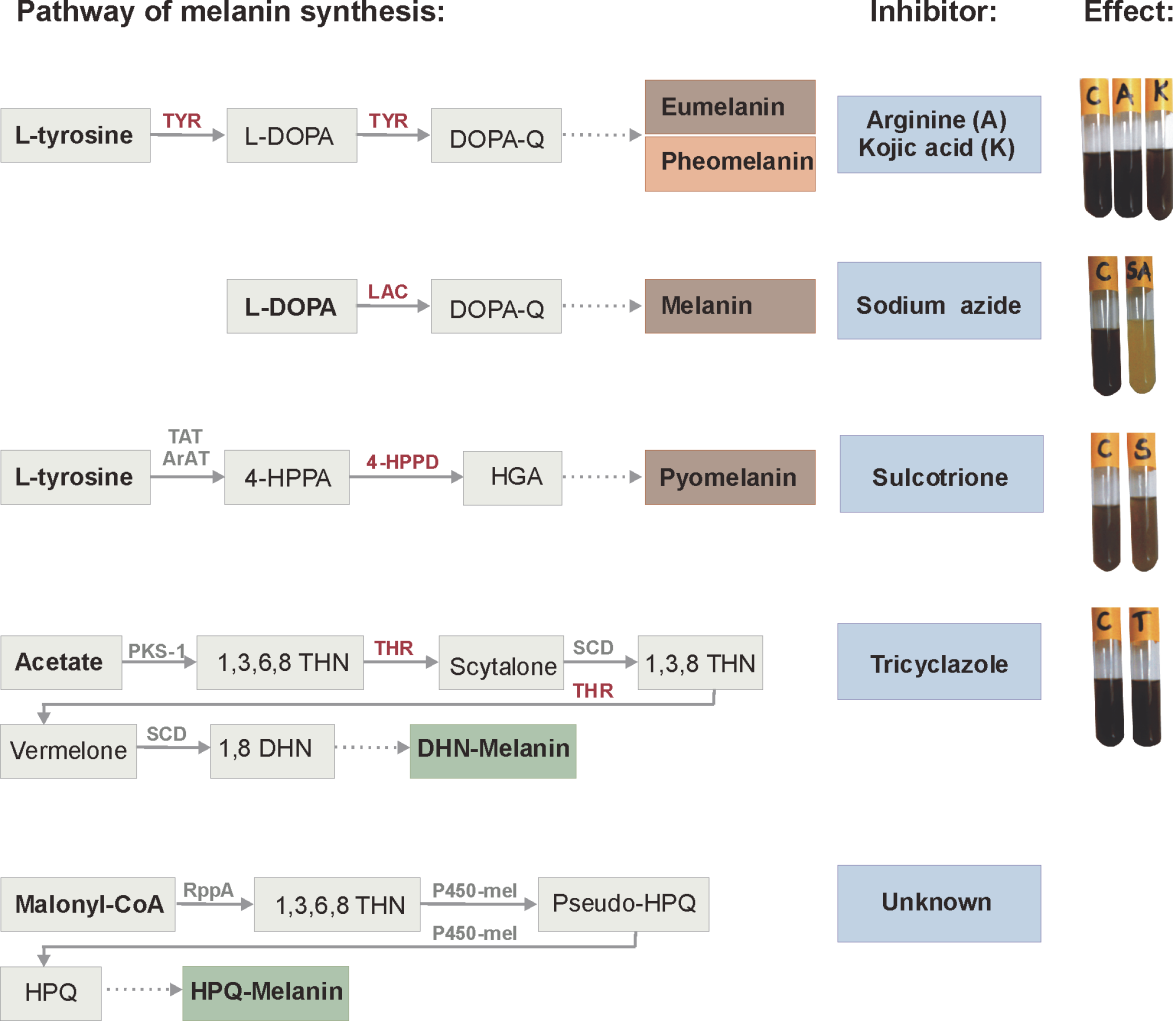
Putative pathways of melanin synthesis and the inhibition tests for pigment production by *B*. *weihenstephanensis* isolates. TYR, tyrosinase (EC 1.14.18.1); L-DOPA, L-3,4-dihydroxyphenylalanine; DOPA-Q, Dopa-quinone; LAC, laccase (EC 1.10.3.2); TAT, tyrosine aminotransferase (EC 2.6.1.5); ArAT, aromatic amino acid aminotransferase (EC 2.6.1.57); 4-HPPA, 4-hydroxyphenylpyruvic acid; 4-HPPD, 4-hydroxyphenylpyruvate dioxygenase (EC 1.13.11.27); HGA, homogentisic acid; PKS-1, polyketide synthase type I; 1,3,6,8 THN, 1,3,6,8-tetrahydroxynaphthalene; THR, Hydroxynaphthalene reductase; SCD, scytalone dehydratase (EC 4.2.1.94); 1,3,8 THN, 1,3,8-trihydroxynaphthalene; 1,8 DHN, 1,8-dihydroxynaphthalene; DHN-Melanin, dihydroxynaphthalene melanin; RppA, polyketide synthase type III; P450-mel, cytochrome P-450 enzyme; HPQ, 1,4,6,7,9,12-hexahydroxyperylene-3,10-quinone; HPQ-Melanin, hexahydroxyperylenequinone melanin; C, control. The dotted line represents the non-enzymatic reactions (oxidation and/or polymerization). Abbreviations of inhibited enzymes are marked in red color.

In a set of biochemical tests, no inhibitory effect on the melanin-like pigment synthesis by environmental *B*. *weihenstephanensis* isolates was observed after supplementation of bacterial culture with arginine and kojic acid (tyrosinase inhibitors), sulcotrione (an inhibitor of 4-hydroxyphenylpyruvate dioxygenase in the HGA pathway) and tricyclazole (an inhibitor of hydroxynaphthalene reductase in the DHN pathway). On the other hand, the addition of 0.1 mM sodium azide, which inhibits laccase, resulted in the inhibition of melanin-like pigment production, indicating that laccase is a key enzyme in the pigment synthesis among *B*. *weihenstephanensis* strains. The addition of higher concentrations of sodium azide resulted in inhibition of bacterial growth. The effects of tested chemicals on melanin-like pigment production in isolates are presented in Fig 5.

## Discussion

Although many microorganisms have been known to synthesize melanin [11–15], *B*. *cereus s*.*l*. species able to produce this pigment are extremely rare in nature [32, 33]. In this study we found only six *B*. *weihenstephanensis* melanin-positive isolates within a *B*. *cereus s*.*l*. collection of about 950 strains of different origin. However, only four strains originated from Jasienowka farm were able to produce melanin-like pigment in/on different media steadily when the experiments were conducted. Chemicals used in agriculture, such as mineral fertilizers or plant protection products, change bacterial communities [52] and may induce mutation(s) altering biochemical pathways associated with melanin synthesis in microorganisms [34, 53]. Melanin producers from Northeastern Poland showed phenotypic and genotypic similarity with psychrotolerant *B*. *weihenstephanensis* DSMZ 11821 reference strain [19]. Within *B*. *cereus* group, the adaptation to low temperatures was regarded to be a characteristic of *B*. *weihenstephanensis* [19]. However, recent studies have indicated that psychrotolerance is also present among other members of the *B*. *cereus* group isolated from Northeastern Poland, one of the coldest region in Poland (Institute of Meteorology and Water Management in Poland, http://www.imgw.pl/klimat/), and revealed the existence of thermal ecotype among soil isolates [37, 41]. *B*. *weihenstephanensis* strains able to grow at low temperatures seem to be perfectly adapted to the habitat they occupy in Northeastern Poland.

The cluster analysis based on biochemical properties, 16S rRNA and housekeeping genes sequences indicated a close relationship among melanin-like pigment producers. A previous study showed that the community of soil *B*. *cereus s*.*l*. strains from Northeastern Poland was genetically diverse, and this diversity mostly resulted from mutation events [37]. Belotte et al. [54] noted that environmental heterogeneity, selectable genetic variation and divergent selection were prerequisites for the emergence of local adaptation. Thus, high similarity of melanin-positive strains derived from genetically polymorphic population indicated that melanogenesis among soil *B*. *weihenstephanensis* might be a local adaptation to specific environmental niches which protects these bacteria from adverse environmental factors, such as UV light [2] extreme temperatures [5] or heavy metals [55]. Similarly, Wang et al. [53] also observed high clonality among natural melanin-producing *Vibrio cholerae* strains and suggested an environmental protective function of melanin in the *V*. *cholerae* community. This raised a question, why bacteria able to produce melanin are very rare in natural environments? We assumed that melanin-like pigment synthesis among *B*. *weihenstephanensis* entails a fitness cost due to the energy expensive pathway involving laccase and/or polymerization of the pigment. Yet, the benefit of extracellular melanin-like pigment production could be shared with co-occurring bacteria which do not synthesize the pigment. Such a cooperation of bacteria to reduce production costs were observed for entomopathogenic toxin synthesis by *B*. *thuringiensis* [56], a close relative of *B*. *weihenstephanensis* [19, 21].

The presence of stable free radicals in all melanin pigments allowed the identification of the pigment as melanin using EPR spectroscopy. The similar shapes of EPR spectra of natural dark particles and the synthetic melanin confirmed the production of melanin pigment by environmental *B*. *weihenstephanensis* isolates. It was shown that o-Semiquinone free radicals (S = 1/2) with unpaired electrons localized on oxygen atoms were responsible for these melanin spectra [57]. Moreover, the typical broadening of melanin spectral curves was caused by dipolar interactions of free radicals in these polymers [58]. FT-IR spectroscopy, which gives precise information on main functional groups of an organic compound, also revealed similar spectra for "environmental" melanin-like pigment produced by soil *B*. *weihenstephanensis* isolates and synthetic melanin, indicating their phenolic character. Some differences in FT-IR spectra of environmental pigment and commercial melanin could result from the procedure of pigment extraction and purification, e.g. the usage of ethanol. According to the actual definition, melanin is a dark in color substance, insoluble in aqueous or organic solvents, resistant to concentrated acid and susceptible to bleaching by oxidizing agents [1]. Physicochemical properties of *B*. *weihenstephanensis* melanin-like pigment and the synthetic one are comparable. It is worth emphasizing that the precipitation with ferric chloride, decolorization by hydrogen peroxide and infrared spectra, indicated the phenolic nature of melanin-like pigments synthesized by environmental *B*. *weihenstephanensis*. The solubility in water of “environmental” melanin was the only difference. Similar results, including also melanin-like pigment water-solubility, were observed by Aghajanyan et al. [34] for *B*. *thuringiensis* subsp. *galleriae* strain K1 obtained by chemical mutagenesis from industrial bioinsecticide *B*. *thuringiensis* 69–6. However, in general melanin produced by bacteria was water-insoluble [32, 59]. Certainly, water-soluble melanin-like pigment as this produced by our *B*. *weihenstephanensis* isolates might have broader biotechnological applications in comparison to the insoluble pigment.

In microbial melanogenesis the most important enzyme seems to be tyrosinase, a monooxygenase which binds two copper ions within the active site by three conserved histidines residues [12]. Indeed, Liu et al. [33] identified the heat-inducible tyrosinase responsible for the pigment production by wild *B*. *thuringiensis* CCTCC AB 90010; however, we did not identify the gene encoding tyrosinase in *B*. *weihenstephanensis* JAS 83/3 genome. Nevertheless, there are several other enzymes potentially involved in melanin production, such as laccase or polyketide synthases [12, 13, 17]. Also some species produce melanin in a way of homogentisic acid polymerization [14, 53], yet little is known about these mechanisms among *B*. *cereus s*.*l*. Here, for the first time, we pointed out that laccase might be involved in the production of melanin-like pigment among strains belonging to the *B*. *cereus* group. While laccase is widely distributed in fungi and plants, in which it can be involved in melanin formation, lignolysis or detoxification, the activity of this enzyme in bacteria has been rarely documented [60, 61]. Laccase enzyme possesses three conservative histidine residues and it has a similar function to tyrosinase, but at the same time, it is activated under different conditions in different species [12]. In fact, the metabolic pathway of melanin synthesis with the participation of laccase is poorly understood and requires further investigation.

## Conclusions

Only a limited number of *B*. *weihenstephanensis* isolates naturally produce melanin-like pigment. This process is probably associated with laccase activity. *B*. *weihenstephanensis* pigment producers from Northeastern Poland demonstrated a high level of phenotypic and genotypic similarity. Our study shed new light on the evolution and adaptation of *B*. *weihenstephanensis* to specific habitats. Because of possible applications, water-soluble melanin-like pigment produced by *B*. *weihenstephanensis* might be an alternative to commercial pigment, thus, it is worth further investigation.

## Supporting Information

S1 Table Phenotypic and genotypic characteristic of melanin-positive *Bacillus weihenstephanensis* isolates and the reference strains. (DOCX)

S2 Table Chemical properties of melanin-like pigment synthetized by soil *B*. *weihenstephanensis* isolates in comparison to reference melanin. (DOCX)
