# Supplementary material for: Melanin-Like Pigment Synthesis by Soil Bacillus weihenstephanensis Isolates from Northeastern Poland
Source: PLoS One. 2015 Apr 24;10(4):e0125428. doi: 10.1371/journal.pone.0125428 (PMC4409349; doi:10.1371/journal.pone.0125428)
Supplement: S1 Table — (DOCX) [file pone.0125428.s001.docx]

Supporting Information Table S1. Phenotypic and genotypic characteristic of melanin-positive *Bacillus weihenstephanensis* isolates and the reference strains.

| **Feature^a^** | **Strain^b^** | | | | | | | | | | | | |
| --- | --- | --- | --- | --- | --- | --- | --- | --- | --- | --- | --- | --- | --- |
|  | *B.w.* JAS 39-1 | *B.w.* JAS 81-4 | *B.w.* JAS 83-3 | *B.w.* JAS 86-1 | *B.w.* BPN 08-1 | *B.w.* BPN 08-4 | *B.w.* DSM 11821 | *B.t.* HD1 | *B.t.* HD73 | *B.t.* HD567 | *B.t.* HD867 | *B.c.* ATCC 10987 | *B.c.* ATCC 14579 |
| Growth at: |  |  |  |  |  |  |  |  |  |  |  |  |  |
| 7^o^C | + | + | + | + | + | + | + |  |  |  |  |  |  |
| 30^o^C | + | + | + | + | + | + | + | + | + | + | + | + | + |
| Nucleotide motif in the *cspA* gene: | | | | | | | | | | | | | |
| Psychrophilic ^4^ACAGTT^9^ | + | + | + | + | + | + | + |  |  |  |  |  |  |
| Mesophilic ^4^GCAGTA^9^ |  |  |  |  |  |  |  | + | + | + | + | + | + |
| Nucleotide motif in the 16S rDNA gene: | | | | | | | | | | | | | |
| Psychrophilic ^1002^TCTAGAGATAGA^1013^ | + | + | + | + | + | + | + |  |  |  |  |  |  |
| Mesophilic ^1002^CCTAGAGATAGG^1013^ |  |  |  |  |  |  |  | + | + | + | + | + | + |
| Parasporal crystal presence |  |  |  |  |  |  |  | + | + | + | + |  |  |
| Fermentation of^c^: |  |  |  |  |  |  |  |  |  |  |  |  |  |
| Glycerol |  |  |  |  |  |  |  | + |  | + | + |  |  |
| D-mannose |  |  |  |  |  |  |  |  |  | + | + | + |  |
| Arbutin | + | + | + | + | + | + | + | + | + |  | + | + | + |
| Salicin | + | + | + | + | + | + | + | + | + |  | + | + | + |
| Cellobiose |  |  |  |  | + | + |  | + | + |  | + | + | + |
| Sucrose | + | + |  | + | + | + |  |  |  |  | + | + | + |
| Arginine dihydrolysis |  |  |  |  | + | + |  |  |  |  |  |  |  |
| Liquefaction of gelatin | + | + | + | + | + | + | + |  |  |  |  | + |  |

^a^ For clarity negative results are omitted.

^b^ JAS, isolates from a farmland in Jasienowka in Northeastern Poland; BPN, isolates from Białowieża National Park; *B.w.*, *B. weihenstephanensis*; *B.t.*, *B. thuringiensis*; *B.c.*, *B. cereus*; *B.w.* DSMZ 11821, *B. weihenstephanensis* DSMZ 11821 type strain (German Collection of Microorganisms and Cell Cultures ); *B.t.* HD1, *B. thuringiensis* HD1 (*Bacillus* Genetic Stock Center, Ohio State University, Columbus, USA, BGSC); *B.t.* HD73, *B. thuringiensis* HD73 (BGSC); *B.t.* HD567, *B. thuringiensis* HD567 (BGSC); *B.t.* HD867, *B. thuringiensis* HD867 (BGSC); *B.c.*  ATCC 10987, *B. cereus* ATCC 10987 (American Type Culture Collection); ATCC 14579, *B. cereus* ATCC 14579 (American Type Culture Collection);

^c^ Only variable results are given. All isolates and the reference strains fermented: ribose, D-glucose, D-fructose, N-acetyloglucosamine, aesculin, maltose, trehalose, glycogen, starch, and gave negative results in the VP test. All isolates and the reference strains were negative for fermentation of: erythritol, D-arabinose, L-arabinose, D-xylose, L-xylose, adonitol, methyl-D-xyloside, galactose, L-sorbose, rhamnose, dulcitol, inositol, mannitol, sorbitol, α methyl-D-mannoside, α methyl-D-glucoside, amygdalin, lactose, melibiose, inuline, melezitose, D-raffinose, xylitol, β-gentiobiose, D-turanose, D-lyxose, D-tagatose, D-fucose, L-fucose, D-arabitol, L-arabitol, gluconate, 2-keto-gluconate, 5-keto-gluconate. In addition the bacilli under study were negative in the following tests: presence of ß-galactosidase (test ONPG), lysine decarboxylation, ornithine decarboxylation, citrate utilization, H_2_S production, presence of urease, tryptophane deamination, and indole production.
