# Supplementary material for: Melanin-Like Pigment Synthesis by Soil Bacillus weihenstephanensis Isolates from Northeastern Poland
Source: PLoS One. 2015 Apr 24;10(4):e0125428. doi: 10.1371/journal.pone.0125428 (PMC4409349; doi:10.1371/journal.pone.0125428)
Supplement: S2 Table — (DOCX) [file pone.0125428.s002.docx]

Supporting Information Table S2. Chemical properties of melanin-like pigment synthetized by soil *Bacillus weihenstephanensis* isolates in comparison to reference melanin.

| **Test** | **Melanin** | |
| --- | --- | --- |
|  | **Produced by *Bacillus weihenstephanensis* isolates** | **Comercial** |
| Color | Blackish-brown | Blackish-brown |
| Solubility in: |  |  |
| Water | + | - |
| 1 N NaOH | + | + |
| Ethanol | - | - |
| Acetone | - | - |
| Chloroform | - | - |
| Benzene | - | - |
| Phenol | + | + |
| Precipitation: |  |  |
| 1N HCl | + | + |
| 1% FeCl_3_ | + | + |
| Decolorization in: |  |  |
| 30% H_2_O_2_ | + | + |
| 5% Na_2_S_2_O_4_ | + | + |
